# Supplementary material for: Complexome profiling of the Chlamydomonas psb28 mutant reveals TEF5 as an early PSII assembly factor
Source: Plant Cell. 2025 Mar 17;37(6):koaf055. doi: 10.1093/plcell/koaf055 (PMC12164590; doi:10.1093/plcell/koaf055)
Supplement: koaf055_Supplementary_Data [file koaf055_supplementary_data.zip › psb28_interactive.zip!/plots/intensity/qualityPlots/peptides/_Cre02.g095077.t1.1.html]

### Cre02.g095077.t1.1

Trivial name:   
SeqCoverage [median]: 44.10  
Mass: 42.93 kDa  
  
 

| Sequence | MissedCleavage | Mass [Da] | wtIntensity | muIntensity | Score |
| --- | --- | --- | --- | --- | --- |
| ATVQEAADIMLK | 0 | 1288.67 | 708.03 | 1278.88 | 133.89 |
| ELLERQDK | 1 | 1029.55 | 3418.26 | 0.00 | 81.30 |
| HMTSPAITVSIR | 0 | 1311.70 | 891.22 | 1757.92 | 93.84 |
| IIFVDVSSSK | 0 | 1093.60 | 9769.30 | 12369.27 | 152.34 |
| ISLGSAMDTLLAGK | 0 | 1375.74 | 4536.82 | 5939.12 | 179.19 |
| ISLGSAMDTLLAGKK | 1 | 1503.83 | 490.87 | 845.52 | 50.56 |
| NMGISQDEAMTTIHSIRPDGSVLQGTDAIR | 1 | 3212.56 | 0.00 | 2072.07 | 40.82 |
| RLPVVDTDGRPLGLVSR | 2 | 1849.05 | 0.00 | 5620.48 | 53.83 |
| SDIFKPLGDYTK | 1 | 1382.71 | 6370.60 | 13773.16 | 90.79 |
| SGSTMTELVK | 0 | 1051.52 | 0.00 | 1181.87 | 82.75 |
| SGSTMTELVKK | 1 | 1179.62 | 640.93 | 1105.80 | 111.63 |
| VAADPSLAQPSTSLEK | 0 | 1612.83 | 2227.28 | 1429.26 | 113.24 |
| VMEEELAMLAGK | 0 | 1319.65 | 3802.25 | 3874.88 | 146.11 |
| VTGLPVLDAEGK | 0 | 1197.66 | 1537.32 | 7385.53 | 103.70 |
| VVGVISR | 0 | 728.45 | 788.07 | 1649.04 | 76.03 |
|  |  |  | 35181.0 | 60282.8 |  |
